# ZFNGenome: A comprehensive resource for locating zinc finger nuclease target sites in model organisms

**DOI:** 10.1186/1471-2164-12-83

**Published:** 2011-01-28

**Authors:** Deepak Reyon, Jessica R Kirkpatrick, Jeffry D Sander, Feng Zhang, Daniel F Voytas, J Keith Joung, Drena Dobbs, Clark R Coffman

**Affiliations:** 1 Department of Genetics, Development and Cell Biology, Iowa State University, Ames IA 50011, USA; 2 Molecular Pathology Unit, Center for Cancer Research, and Center for Computational and Integrative Biology, Massachusetts General Hospital, Charlestown, MA 02129, USA; 3 Department of Pathology, Harvard Medical School, Boston, MA 02115, USA; 4 Department of Genetics, Cell Biology, and Development, and Center for Genome Engineering, University of Minnesota, Minneapolis, MN 55455, USA; 5 Cellectis Plant Sciences, 1000 Westgate Drive, St. Paul, MN 55114, USA; 6 Bioinformatics and Computational Biology Graduate Program, Iowa State University, Ames IA 50011, USA

## Abstract

**Background:**

Zinc Finger Nucleases (ZFNs) have tremendous potential as tools to facilitate genomic modifications, such as precise gene knockouts or gene replacements by homologous recombination. ZFNs can be used to advance both basic research and clinical applications, including gene therapy. Recently, the ability to engineer ZFNs that target any desired genomic DNA sequence with high fidelity has improved significantly with the introduction of rapid, robust, and publicly available techniques for ZFN design such as the Oligomerized Pool ENgineering (OPEN) method. The motivation for this study is to make resources for genome modifications using OPEN-generated ZFNs more accessible to researchers by creating a user-friendly interface that identifies and provides quality scores for all potential ZFN target sites in the complete genomes of several model organisms.

**Description:**

ZFNGenome is a GBrowse-based tool for identifying and visualizing potential target sites for OPEN-generated ZFNs. ZFNGenome currently includes a total of more than 11.6 million potential ZFN target sites, mapped within the fully sequenced genomes of seven model organisms; *S. cerevisiae, C. reinhardtii, A. thaliana*, *D. melanogaster, D. rerio, C. elegans*, and *H. sapiens *and can be visualized within the flexible GBrowse environment. Additional model organisms will be included in future updates. ZFNGenome provides information about each potential ZFN target site, including its chromosomal location and position relative to transcription initiation site(s). Users can query ZFNGenome using several different criteria (e.g., gene ID, transcript ID, target site sequence). Tracks in ZFNGenome also provide "uniqueness" and ZiFOpT (Zinc Finger OPEN Targeter) "confidence" scores that estimate the likelihood that a chosen ZFN target site will function *in vivo*. ZFNGenome is dynamically linked to ZiFDB, allowing users access to all available information about zinc finger reagents, such as the effectiveness of a given ZFN in creating double-stranded breaks.

**Conclusions:**

ZFNGenome provides a user-friendly interface that allows researchers to access resources and information regarding genomic target sites for engineered ZFNs in seven model organisms. This genome-wide database of potential ZFN target sites should greatly facilitate the utilization of ZFNs in both basic and clinical research.

ZFNGenome is freely available at: http://bindr.gdcb.iastate.edu/ZFNGenome or at the Zinc Finger Consortium website: http://www.zincfingers.org/.

## Background

The ability to efficiently modify the genome of an organism with a high degree of specificity would advance both research with model organisms and human gene therapy clinical trials [1-3]. In recent studies, zinc finger nuclease (ZFN)-mediated genomic modification rates of 3% - 100% for specific genes have been reported in zebrafish, *Arabidopsis*, and rat [4-16]. Moreover, ZFNs are being evaluated in human gene therapy clinical trials for treating AIDS [11,17-19]. Thus, ZFNs are emerging as premier tools for site-specific genomic modification in both animals and plants.

**Figure 1:**
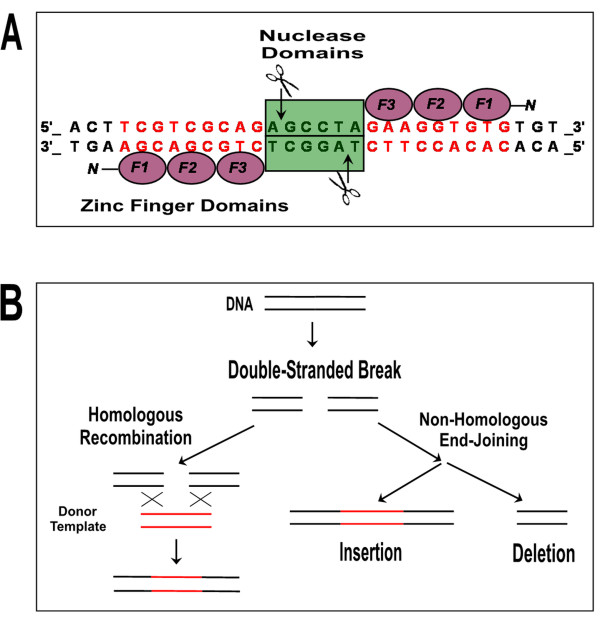
**ZFNs generate site-specific double-stranded breaks that can be used for homologous recombination or mutagenesis**. (A) ZFNs are composed of two arrays that recognize 9-12 base pairs each. Two arrays with three fingers, F1-F2-F3, that recognize nine base pairs each are shown. Each array is fused to one half of a nonspecific *Fok*I endonuclease (green). Upon dimerization, the *Fok*I endonuclease is activated and creates a double-stranded break at sites flanked by the DNA binding sites recognized by the zinc finger arrays. Scissors and arrows denote the cut sites. (B) In most cells, double-stranded breaks (DSBs) are repaired by one of two major pathways. If a donor template is available, homologous recombination can result in engineered nucleotide substitutions at the target site (left). Alternatively, DSBs can be repaired by non-homologous end-joining, an error-prone mechanism that frequently results in small deletions or insertions at the site of the DSB (right).

Engineered ZFNs consist of two zinc finger arrays (ZFAs), each of which is fused to a single subunit of a non-specific endonuclease, such as the nuclease domain from the *Fok*I enzyme, which becomes active upon dimerization [20,21]. Typically, a single ZFA consists of 3 or 4 zinc finger domains, each of which is designed to recognize a specific nucleotide triplet (GGC, GAT, etc.) [22]. Thus, ZFNs composed of two "3-finger" ZFAs are capable of recognizing an 18 base pair target site; an 18 base pair recognition sequence is generally unique, even within large genomes such as those of humans and plants. By directing the co-localization and dimerization of two FokI nuclease monomers, ZFNs generate a functional site-specific endonuclease that creates a double-stranded break (DSB) in DNA at the targeted locus [23] (Figure 1A).

In eukaryotes, repair of DSBs in DNA is primarily accomplished via one of two pathways, homologous recombination (HR) and non-homologous end-joining (NHEJ) (Figure 1A). Depending on the desired modification, either pathway can be exploited in ZFN-mediated genomic engineering. Because HR relies on homologous DNA to repair the DSB, gene targeting can be achieved by supplying an exogenous "donor" template. This results in replication of the "donor" DNA sequence at the target locus, a process that has been utilized to introduce small mutations or large insertions [4,9,12,13,16,24-27]. In contrast, NHEJ is an error-prone repair process and hence is ideal for generating mutations that can result in gene knockouts or knock-downs when the ZFN-mediated DSB is introduced into the protein coding sequence of a gene [5-9,11,28,29].

Oligomerized Pool Engineering (OPEN) is a highly robust and publicly available protocol for engineering zinc finger arrays with high specificity and *in vivo *functionality [9,30,31]. OPEN has been successfully used to generate ZFNs that function efficiently in plants [13,15], zebrafish [6], and human somatic [9] and pluripotent stem cells [16]. OPEN is a selection-based method in which a pre-constructed randomized pool of candidate ZFAs is screened to identify those with high affinity and specificity for a desired target sequence. Significantly higher *in vivo *success rates have been reported using OPEN-generated ZFNs, compared with ZFNs generated using the more traditional modular assembly approach [32-34]. Resources for generating ZFNs using OPEN have been developed and made publicly available by the Zinc Finger Consortium [9,31,35]. Currently, OPEN reagents include modules that recognize all 16 possible GNN triplets (i.e., DNA triplets beginning with G, followed by any nucleotide in the second and third positions), as well as several TNN triplets. Thus, all DNA sites that contain only GNN and/or select TNN triplets can potentially be targeted using the OPEN protocol [9].

To facilitate use of OPEN ZFNs for genome modification, we have developed *ZFNGenome*, a resource that displays potential ZFN target sites in a genome browser built on the user-friendly GBrowse platform [36]. We analyzed the complete sequenced genomes of seven model organisms and identified all sequences that are potentially targetable using currently available OPEN ZFN reagents. ZFN reagents were obtained from Joung and colleagues [9], and ZFN target sites were identified using software implemented in the ZiFiT web server [37,38]. ZFNGenome thus allows users to quickly evaluate "pre-identified" ZFN target sites for any desired gene or region of interest.

To our knowledge, ZFNGenome represents the first compendium of potential ZFN target sites in sequenced and annotated genomes of model organisms. The current version includes ZFN target sites in seven organisms: *Saccharomyces cerevisiae *(budding yeast), *Chlamydomonas reinhardtii *(green algae), *Arabidopsis thaliana *(thale cress), *Caenorhabditis elegans *(nematode), *Drosophila melanogaster *(fruit fly), *Danio rerio *(zebrafish), and *Homo sapiens *(human). Additional model organisms, including three plant species; *Glycine max *(soybean), *Oryza sativa *(rice), *Zea mays *(maize), and three animal species *Tribolium castaneum *(red flour beetle), *Mus musculus *(mouse), *Rattus norvegicus *(brown rat) will be added in the near future.

## Construction and Content

**Figure 2:**
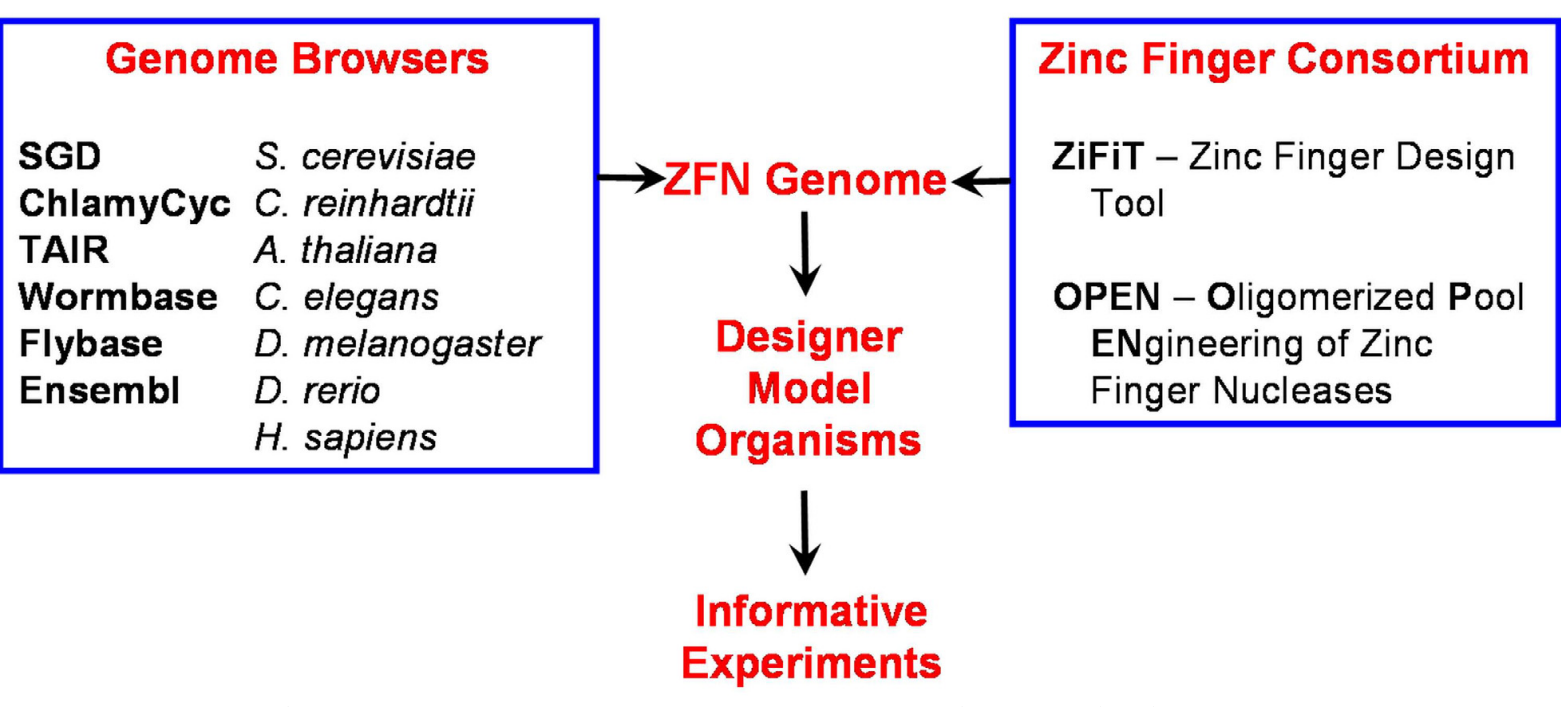
**An overview of the ZFNGenome architecture**. ZFNGenome creates a user-friendly interface for visualizing all potential ZFN target sites in seven model organisms by integrating the genomic information from genome browsers of sequenced and annotated genomes with the tools of the Zinc Finger Consortium. This interface allows researchers using these model organisms to easily determine whether ZFNs are available for the design and execution of targeted genome modifications.

The motivation for implementing ZFNGenome, summarized in Figure 2, was to create a user-friendly interface between two valuable open-source genomic resources: i) established genome browsers, with associated genomic DNA sequences, annotations and other resources available for model organisms; and ii) ZFN design software tools and experimental reagents made available by the Zinc Finger Consortium. ZFNGenome integrates these resources by allowing users to visualize all potential ZFN target sites in a chosen gene or genomic region of a sequenced model organism, with flexible viewing options and annotated genomic features provided in a GBrowse interface.

Table 1 lists the organisms for which complete genomic sequence data were analyzed in this study, along with the data sources for genomic DNA sequences and annotations. The number of potential OPEN target sites identified is shown for each organism. To identify all potential OPEN ZFN target sites, annotated complete genome sequence files were scanned using the ZiFiT algorithm [38], which was modified to accommodate the sequences of an entire chromosome. Only sites for which ZFNs can be engineered using currently available OPEN reagents and spacer distances between the two ZFAs of 5, 6 or 7 base pairs were included [9]. Because OPEN selections are performed in a Dam+/Dcm+ *E. coli *strain, genomic target sequences that contain potential *dam *or *dcm *methylation sites were excluded from consideration, as were sites that lacked a GNN subsite. Previous studies have shown that most successful OPEN sites contain at least one GNN [31].

**Table 1:** Model organism genomes analyzed and the number of OPEN ZFN target sites identified

Organism	**Source **^**1**^	Total # of OPEN **target sites**^**2**^	Total # of **transcripts**^**2**^	ZFN targetable **transcripts**^**2**^		Avg. # ZFN target sites **per transcript**^**2**^	GC Content
				**#**	**%**		

*Saccharomyces cerevisiae*	SGD	31,822	6,685	5,810	87	5.5	38.3

*Chlamydomonas reinhardtii*	ChlamyCyc	330,136	15,496	14,423	93	22.9	58.1

*Arabidopsis thaliana*	TAIR	171,409	33,200	30,193	91	5.7	35.5

*Caenorhabditis elegans*	WormBase	112,725	28,202	23,861	85	4.7	34.2

*Drosophila melanogaster*	FlyBase	185,863	21,736	20,259	93	9.2	40.9

*Danio rerio*	Ensembl	214,809	27,305	25,918	95	8.3	35.9

*Homo sapiens*	Ensembl	670,597	71,913	66,170	92	10.1	37.1

^1 ^Data Source URLs: SGD - http://www.yeastgenome.org/

ChlamyCyc - http://chlamyto.mpimp-golm.mpg.de/chlamycyc/index.jsp

TAIR - http://www.arabidopsis.org/

WormBase - http://www.wormbase.org/

FlyBase - http://flybase.org/

Ensembl *Danio rerio *- http://uswest.ensembl.org/Danio_rerio/Info/Index

Ensembl *Homo sapiens*- http://uswest.ensembl.org/Homo_sapiens/Info/Index

^2 ^"Transcripts" refers to protein encoding transcripts mapped onto chromosomes (i.e., scaffolds are not included).

Note: For *Chlamydomonas reinhardtii *the average number of ZFN target sites per transcript is very high. This likely reflects the increased GC content of this genome.

ZFNGenome utilizes GBrowse 1.7 [36] to display identified potential OPEN target sites, along with basic genome annotations, such as genes, transcripts, exons, introns, and 5' and 3' UTRs. ZFNGenome is hosted on an Apache2 web server and uses a MySQL DB linked to a GBrowse front end via open source adaptors available in BioPerl (version 1.6) [39]. The ZFN target sites can be exported for use as annotations in other GBrowse-based genome browsers such TAIR and WormBase. As described below, each ZFN target site is hyperlinked to ZiFDB [40].

### Resources available in ZFNGenome

**Figure 3:**
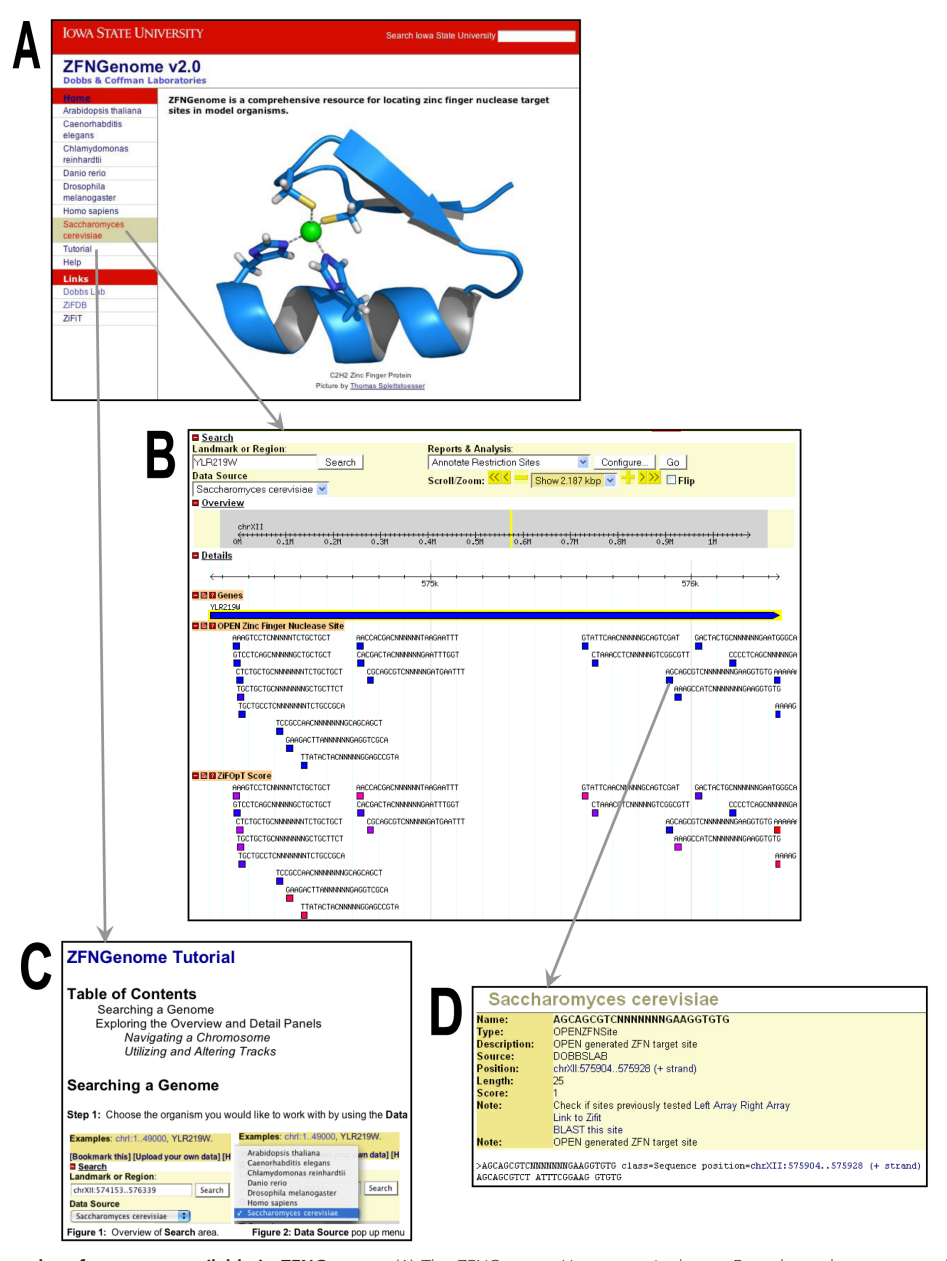
**Examples of resources available in ZFNGenome**. (A) The ZFNGenome Homepage is shown. From here, the user can select a model organism from the seven shown in the left hand column. In addition, links to the ZFNGenome *Tutorial *and *Help *pages are provided. (B) A screenshot of the result of a search of the *S. cerevisiae *gene YLR219W is displayed. Key areas of the browser include the search box and the "Scroll/Zoom" areas at the top. The "Overview" and "Detail" panels serve as controls for visualizing the genome. This search shows the single coding region of this gene has 17 potential ZFN target sites, color-coding according to their "uniqueness" and "ZiFOpT" scores (see text). Additional information on each of the tracks can be obtained by clicking on details of the track. For example, clicking on one of the OPEN Zinc Finger Nuclease Sites links the user to details about that specific ZFN target. (C) The ZFNGenome *Tutorial *offers instructions on navigating the database. The *Tutorial *can be accessed from the Homepage or from any GBrowse page within ZFNGenome. *Help *and *Instruction *links are provided from the GBrowse pages. (D) Clicking on a ZFN target site opens a new window that provides links to ZiFDB, which provides additional information for each zinc finger array, ZiFiT the zinc finger design software that includes the OPEN design method and zinc finger pools, and the BLAST server at National Center for Biotechnology Information (NCBI).

Users can choose the model organism of interest from the ZFNGenome homepage, http://bindr.gdcb.iastate.edu/ZFNGenome, by choosing an organism from the left hand column of the front page or via the "Data Source" dropdown menu from within an organism's ZFNGenome page (Figures 3A and 3B). Figure 3B is a screenshot of the output displayed in response to a search for ZFN target sites in the *Saccharomyces cerevisiae *genome. Several standard GBrowse tracks are displayed by default (genes, transcripts, coding regions, etc.). The *OPEN Zinc Finger Nuclease Sites *track shows that within the 2.187 kb region illustrated (gene YLR219W) there are 17 potential ZFN target sites located in the coding region of this gene. A "uniqueness score" is reflected in the color-coding of ZFN target sites in this track: blue sites are unique; purple sites are present in 2-9 copies; red sites are present in more than 9 copies within the genome of the organism displayed. Because OPEN reagents are available to recognize all possible GNN and some TNN triplets, we have included a track illustrating analysis of the GC content of the DNA. Clicking on any genomic feature illustrated below the sequence reveals additional information about that feature. For example, in the *OPEN Zinc Finger Nuclease Site *track, clicking on (AGCAGCGTCNNNNNNNGAAGGTGTG) opens a page containing more information about the site, as illustrated in Figure 3D. The "Note" sections on this page provide links to ZiFDB [40], a repository for zinc finger arrays that have been experimentally validated, and ZiFiT [37], a tool for identifying potential ZFP and ZFN target sites. A hyperlink to NCBI BLAST [41] can be used to examine additional ZFN sites (if any) in the genome of interest. The *ZiFOpT Score *track is provided to help users rank potential ZFN target sites according to the likelihood that they will function successfully. The ZiFOpT score is based on a naïve Bayes classifier that predicts whether or not a given ZFN site will be active *in vivo *[42]. Potential ZFN target sites are also color-coded in the *ZiFOpT Score *track: blue sites are most likely to be active *in vivo*; purple sites are less likely to be active; red sites are predicted to be inactive. By clicking on features in other tracks, an investigator can access the exact sequence, chromosomal position and sources of reagents needed to experimentally target the chosen site. Users may customize the GBrowse display by choosing which feature(s) to display (using the -/+ buttons on the left), and defining the order in which features are displayed by dragging and dropping the features within the browser window. The ZFN tracks can be exported back into the "home" GBrowse website for a model organism by clicking on the "share the track" button (details provided in the *Tutorial*, Figure 3C). Users can also utilize *Help, Instruction*, and *Tutorial *functions within the browser windows to obtain more information about navigating ZFNGenome.

To evaluate the reliability of data presented in ZFNGenome, we compared our results with other published data. Two types of data are presented in ZFNGenome: annotated genomic features and potential ZFN target sites. The sources from which we acquired the genomic features are listed in Table 1. These are widely considered to be the "gold standard" data sources for the model organisms analyzed because they are carefully annotated and repeatedly evaluated by the curators and users of these databases. These source databases are also extensively used by investigators utilizing the various model organisms and are therefore familiar to users. To identify potential errors that may have been introduced during pre-processing or data analysis, we performed quality assurance tests as follows: i) for each organism, several 5 kb segments of genomic sequence were randomly selected from each chromosome; 2) selected chromosomal DNA sequences were individually re-scanned using the ZiFiT web server [37] to identify potential OPEN ZFN sites; 3) sites identified by the ZiFiT server were directly compared to the results for the corresponding region obtained from the ZFNGenome database; genomic features were checked against the original database. To improve the user interface and documentation, we incorporated suggestions from at least one expert scientist for each of model organisms included in ZFNGenome.

## Utility and Discussion

### Currently available ZFNs can target 80 - 95% of protein coding transcripts in 7 model organisms

The results presented in Table 1 illustrate both the power and current limitations of OPEN ZFN engineering technology and identify gaps where further improvement is needed. Most striking is the relatively high level of coverage currently achievable. This ranges from 85% of protein coding transcripts in *Caenorhabditis elegans *to 95% of protein coding transcripts in *Danio rerio. *Also noteworthy is the number of potential target sites available within any given transcript: in the model organisms examined to date, each transcript contains, on average 5 - 23 target sites (Table 1). The current lack of OPEN ZFN reagents for targeting TNN, ANN and CNN triplets is a limitation, especially in organisms with AT rich genomes. However, even in Arabidopsis (35.5% GC) more than 91% of the protein coding transcripts are potentially targetable. As more ZFN reagents for targeting additional triplets become available, the applicability of ZFN technology will continue to increase.

The first study in which the entire genome of a model organism was analyzed to identify potential target sites for ZFNs focused on the zebrafish, *Danio rerio *[6]. In that study, identified ZFN target sites were published in the form of 26 supplemental tables (one for each chromosome). Although this information has apparently proven useful for members of the zebrafish community, ZFNGenome was developed in an effort to make such large datasets searchable and more readily accessible to a broader group of researchers working in zebrafish as well as other model organisms.

Because the experimental generation and testing of ZFNs using the OPEN protocol is not a trivial undertaking, the utility of a method to discriminate between ZFN target sites that are likely to function successfully *in vivo *and those that are not, cannot be over-emphasized. Our analysis discussed above reveals that, on average, every transcript in the zebrafish genome contains ~ 8 potential ZFN target sites (see Table 1). In ZFNGenome, the incorporation of "uniqueness" and ZiFOpT "confidence" scores (42) should help improve the time and cost-effectiveness of genomic modification experiments utilizing ZFNs.

In the first implementation of ZFNGenome, we used GBrowse version 1.67 with a BerkeleyDB back end to display all potential ZFN target sites found in *Arabidopsis *[15]. A total of 381,497 sites were identified, 171,409 of which were located within coding regions (an average of 5.7 sites per targetable transcript). The current version of ZFNGenome (2.0) includes *S. cerevisiae*, *C. reinhardtii*, *A. thaliana*, *C. elegans*, *D. melanogaster*, *D. rerio*, and *H. sapiens*. In addition, it has been implemented in the newer GBrowse 1.7 with a MySQL database, which results in a more dynamic and user-friendly interface. GBrowse 1.7 is a robust and highly customizable browser available from the Generic Model Organism Database project (GMOD) [36]. A noteworthy feature is the ability to share tracks with other GBrowse-based resources. To date ~119 implementations of GBrowse are available http://gmod.org/wiki/GMOD_Users. Users accustomed to using popular model organism resources, such as TAIR for Arabidopsis [43] or FlyBase for Drosophila [44], can simply export tracks containing ZFN target sites from ZFNGenome and into their browser of choice for further analysis.

### Related Resources

Several existing databases house information on ZFPs and associated binding sites. ZiFDB http://bindr.gdcb.iastate.edu/ZiFDB contains information about engineered zinc finger arrays and individual modules that have been experimentally evaluated for function *in vivo *[40]. ZifBase http://web.iitd.ac.in/~sundar/zifbase/ is a repository that includes information about both naturally occurring and engineered zinc finger proteins [45]. Sequences of ZFP binding sites are also collected in TRANSFAC [46]http://www.biobase-international.com/index.php?id=transfac and JASPAR http://jaspar.genereg.net/[47]. Tools for predicting the DNA target sites for a selected ZFP include ZIFIBI http://bioinfo.hanyang.ac.kr/ZIFIBI/frameset.php, a hidden Markov model based predictor that takes into account the interdependence between positions -1, +3 and +6 of a chosen ZFP to predict its potential DNA binding site(s) [48]. Also, Persikov et al. [49] have used support vector machines (SVMs) to predict and rank potential ZFP binding sites for a selected ZFP.

Several web-based tools for identifying potential ZFN binding sites within a given DNA sequence are currently available. Zinc Finger Tools http://www.scripps.edu/mb/barbas/zfdesign/zfdesignhome.php can be used to identify target sites for zinc finger arrays composed of available modules (16 GNN, 15 ANN, 15 CNN), generated by the Barbas laboratory, within any given DNA sequence up to 10 kb in length [50]. ZifBase tools http://web.iitd.ac.in/~sundar/zifbase/ can identify target sites in a given DNA sequence, with the option of using target site triplet composition (i.e., the number of GNN, CNN, TNN and ANN triplets) as a selection criterion. TagScan http://www.isrec.isb-sib.ch/tagger/tagscan.html is capable of performing searches for either exact or nearly exact matches (≤ 2 mismatches) between a given query sequence, such as a ZFP target site, and a large database, such as a genomic sequence database [51]. ZiFiT http://bindr.gdcb.iastate.edu/zifit/ is similar to ZFTools in that it allows users to identifying target sites for ZFNs. ZiFiT also can identify sites potentially targetable with ZFPs made from zinc finger modules developed and/or characterized by the Barbas lab, Sangamo BioSciences, Inc., and Toolgen http://www.toolgen.com.

In contrast to all of these existing web-based tools, which identify potential ZFN target sites within a user-provided DNA sequence (typically < 10 kb), ZFNGenome is a comprehensive repository that contains all potential ZFN sites targetable using available OPEN reagents in the complete genomic sequences of 7 model organisms. To help users distinguish between high and low quality potential ZFN target sites, ZFNGenome provides two metrics: a "uniqueness" score showing the number of times a sequence is found within the given genome and a ZiFOpT score providing a prediction of the likelihood that a given ZFN will be active *in vivo*.

### Planned future development

ZFNGenome will be updated regularly to incorporate revisions in genomic DNA sequences and annotations, and to take into account new potential ZFN target sites that can be considered when new reagents, such as additional OPEN pools, become available. The genomes of several other established and emerging model organisms currently in the pipeline include: maize, rice, soybean, red flower beetle, mouse, and rat. We also intend to implement additional features, including capabilities for identifying target sites for ZFNs made by other publicly available engineering methods such as modular assembly.

## Conclusions

OPEN is a robust, publicly available, experimental platform for the generation of engineered ZFNs that function with high specificity *in vivo*. ZFNGenome was developed to enhance and broaden the applicability of ZFNs for genomic modification by providing an online resource that contains all potential target sites for OPEN-generated ZFNs in the sequenced genomes of several model organisms. ZFNGenome has a user-friendly interface and is seamlessly integrated with other publicly available Zinc Finger Consortium resources, such as ZiFiT, ZiFDB, and ZiFOpT. ZFNGenome should be a valuable resource for scientists and clinicians who wish to exploit the powerful technologies for genome modification now available as a result of recent developments in ZFP design and engineering.

## Availability and Requirements

ZFNGenome is freely available over the web at http://bindr.gdcb.iastate.edu/ZFNGenome or through "Software Tools" at the Zinc Finger Consortium website: http://www.zincfingers.org/.

## List of Abbreviations Used

OPEN: Oligomerized Pool ENgineering; ZF: Zinc Finger; ZFA: Zinc Finger Array; ZFP: Zinc Finger Protein; ZFN: Zinc Finger Nuclease; ZiFOpT: Zinc Finger OPEN Targeter; DSB: double stranded breaks; HR: homologous recombination; NHEJ: non-homologous end joining; GMOD: generic model organism database project; SVMs: support vector machines; ZiFDB: Zinc Finger Database

## Authors' contributions

All authors contributed to the overall concept and DR directed the design and implementation of the database. DR and JK identified ZFN target sites in model organism genomes, constructed the database, provided online documentation, and designed the web interface. JK, DR, and CRC performed quality control assessment. DR, JK, DD and CRC drafted the manuscript. All authors read, contributed to revisions of and approved the final manuscript.
